# tRNA^Lys^-Derived Fragment Alleviates Cisplatin-Induced Apoptosis in Prostate Cancer Cells

**DOI:** 10.3390/pharmaceutics13010055

**Published:** 2021-01-04

**Authors:** Changwon Yang, Minkyeong Lee, Gwonhwa Song, Whasun Lim

**Affiliations:** 1 Department of Biotechnology, Institute of Animal Molecular Biotechnology, College of Life Sciences and Biotechnology, Korea University, Seoul 02841, Korea; ycw117@korea.ac.kr; 2 Department of Food and Nutrition, College of Science and Technology, Kookmin University, Seoul 02707, Korea; m2019546@kookmin.ac.kr

**Keywords:** prostate cancer, tRNA-derived fragments, cisplatin, apoptosis, *GADD45A*

## Abstract

Cisplatin is a standard treatment for prostate cancer, which is the third leading cause of cancer-related deaths among men globally. However, patients who have undergone cisplatin can rxperience relapse. tRNA-derived fragments (tRFs) are small non-coding RNAs generated via tRNA cleavage; their physiological activities are linked to the development of human diseases. Specific tRFs, including tRF-315 derived from tRNA^Lys^, are highly expressed in prostate cancer patients. However, whether tRF-315 regulates prostate cancer cell proliferation or apoptosis is unclear. Herein, we confirmed that tRF-315 expression was higher in prostate cancer cells (LNCaP, DU145, and PC3) than in normal prostate cells. tRF-315 prevented cisplatin-induced apoptosis and alleviated cisplatin-induced mitochondrial dysfunction in LNCaP and DU145 cells. Moreover, transfection of tRF-315 inhibitor increased the expression of apoptotic pathway-related proteins in LNCaP and DU145 cells. Furthermore, tRF-315 targeted the tumor suppressor gene *GADD45A*, thus regulating the cell cycle, which was altered by cisplatin in LNCaP and DU145 cells. Thus, tRF-315 protects prostate cancer cells from mitochondrion-dependent apoptosis induced by cisplatin treatment.

## 1. Introduction

Prostate cancer is the third leading cause of cancer-related death among men worldwide [1]. Initially, most patients with prostate cancer respond to androgen-deprivation therapies; however, the tumor gradually becomes androgen-independent. Platinum-based anticancer drugs, including cisplatin, are used to treat prostate cancer; however, long-term cisplatin treatment leads to the development of resistance in patients [2]. Cisplatin works by binding to DNA and, in prostate cancer, it inhibits cell growth and induces apoptosis in both a P53-dependent and -independent manner [3,4]. Several pieces of research have shown that different types of non-coding RNAs (ncRNAs) are involved in the progression of prostate cancer and can be used to predict the prognosis for chemotherapy [5,6].

ncRNAs are not translated into proteins but have a wide range of biological functions in organisms. Among ncRNAs, small non-coding RNAs (sncRNAs), which are shorter than 40 nucleotides in length and include micro RNAs (miRNAs), contribute to genetic regulation through complementary binding to DNA and are associated with the development of several human diseases, including cancer [7]. In addition, studies over the past 20 years have demonstrated the possibility of using sncRNAs as biomarkers for the early diagnosis of cancer [8]. Moreover, sncRNAs regulate the sensitivity of cancer cells to anticancer agents [9,10,11].

tRNA-derived RNA fragments (tRFs) are sncRNAs that are derived from the 5′ or 3′ end of mature or precursor tRNAs [12]. Recent advances in sequencing technology have facilitated the determination of the expression profiles of tRFs in various cancers, including breast, colon, pancreatic, and prostate cancers [13,14,15]. Compared to other studies that have reported the tRF profiles of other carcinomas, studies on prostate cancer have progressed from the determination of tRF expression profiles to the use of tRFs therapeutically or diagnostically [12,16,17]. Several tRFs, which are dependent on the action of androgen and its receptor, promote the proliferation of prostate cancer cells [18]. Moreover, RNA sequencing analysis performed by Olvedy et al. on clinical samples of patients with prostate cancer suggests that tRF-315 derived from tRNA^Lys^, tRF-562 derived from tRNA^Gly^, and tRF-544 derived from tRNA^Phe^ may serve as markers for predicting the recurrence of prostate cancer [16]. Particularly, the ratio of tRF-315 to tRF-544 is considered to be an effective indicator of progression-free survival in patients with prostate cancer. However, the role of tRFs, whose clinical utility in prostate cancer has been proven, in the regulation of the growth of prostate cancer cells and in cisplatin sensitivity remains to be elucidated.

Therefore, this study confirmed the effect of tRF-315, derived from tRNA^Lys^, on the progression of prostate cancer. The expression of tRF-315, which was previously reported to be high in patients with prostate cancer, in normal prostate cells was compared with that in prostate cancer cell lines. In addition, it was confirmed that the proliferation and apoptosis of prostate cancer cells were regulated in response to tRF-315 expression and cisplatin treatment. Moreover, we investigated whether tRF-315 expression and cisplatin treatment were involved in the regulation of mitochondrial membrane potential in prostate cancer cells. As a result, we verified that tRF-315 does not affect the proliferation of prostate cancer cells, but can contribute to mitochondrial dysfunction and apoptosis, with the regulation of target genes.

## 2. Materials and Methods

### 2.1. Cell Culture

All cell lines were purchased from the American Type Culture Collection. WPMY1 cells were cultured in Dulbecco’s Modified Eagle Medium (DMEM) (Hyclone, Carlsbad, CA, USA) supplemented with 5% fetal bovine serum (FBS). RWPE1 cells were cultured in Keratinocyte Serum Free Medium (Gibco, Waltham, MA, USA) supplemented with 0.05 mg/mL bovine pituitary extract and 5 ng/mL epidermal growth factor. PC3, DU145, and LNCaP cells were cultured in RPMI-1640 medium (Hyclone) supplemented with 10% FBS and 1% penicillin/streptomycin (100 U/mL). Cisplatin (cis-diamminedichloroplatinum) was purchased from Sigma-Aldrich (St. Louis, MO, USA).

### 2.2. Detection of tRFs

Total RNA was extracted from cells using Trizol (Invitrogen, Carlsbad, CA, USA). Previously reported miRNA detection methods, involving the use of digoxygenin (DIG)-labeled DNA probes, splinted ligation, and 1-ethyl-3-(3-dimethylaminopropyl)carbodiimide hydrochloride (EDC) cross-linking, were used to detect tRFs from total RNA, with modifications [19]. A DIG-labeled probe is a fixed sequence in which the phosphate is conjugated at the 5′ end and DIG is conjugated at the 3′ end. A bridge oligonucleotide is a long DNA sequence that is complementary to both the DIG-labeled probe and tRFs. The sequences of the DIG-labeled probe used in the experiment and that of the bridge oligonucleotides used for tRF-315 and miR-21 are as follows: DIG-labeled probe, 5′-(Phosphate)CGC TTA TGA CAT TC(DIG)-3′; tRF-315 bridge, 5′-GAA TGT CAT AAG CGC CAT GCT CTA CCG ACT GAG CTA GCC GGG C-3′; miR-21 bridge, 5′-GAA TGT CAT AAG CGT CAA CAT CAG TCT GAT AAG CTA-3′. All oligonucleotides were synthesized by Bioneer (Daejeon, Korea). Equal concentrations (0.1 μM) of bridge oligonucleotide and probe were mixed with total RNA. Next, T4 DNA ligase was added to the mixture to ligate the nicks and the mixture was incubated at 30 °C for 1 h. The ligation reaction mixture supplemented with RNA loading buffer was loaded on a 12.5% urea-polyacrylamide gel and electrophoresed at 200 V for 30 min. Later, the RNA was transferred onto nylon membranes and the membranes were then incubated with a crosslinking solution containing 1-methylimidazole (Sigma-Aldrich) and EDC (Sigma-Aldrich) for 2 h at 60 °C. DIG Wash and Block Buffer Set (Roche, Indianapolis, IN, USA) were used to detect the DIG signals.

### 2.3. Quantitative RT-PCR for tRFs

The miRNA 1st-strand cDNA Synthesis Kit (Agilent Technologies, Santa Clara, CA, USA) was used to perform polyadenylation reactions and cDNA synthesis using total RNA. Later, miRNA qPCR Master Mix (Agilent Technologies) was used to measure the expression of tRFs. The sequence of the primer used for PCR is as follows: tRF-315, 5′-GCC CGG CTA GCT CAG TCG GTA GAG CAT GG-3′. We determined the level of expression of tRF-315 using standard curve method and cycle threshold (C_T_) values, and U6 sncRNA expression was used to normalize the expression of tRF-315. Relative quantification of tRF-315 was analyzed using the 2^−ΔΔCT^ method.

### 2.4. Transfection

The mimic and inhibitor for tRF-315 was transfected into prostate cancer cells using Lipofectamine 2000. Briefly, cells (4 × 10^5^ cells) were seeded onto 6-well plates. Then, the cells were cultured in Opti- Minimum Essential Media (MEM) reduced serum medium (Gibco) containing mimic and inhibitor for tRF-315 and Lipofectamine 2000. After incubation for 6 h at 37 °C, the cells were treated with cisplatin. The sequence of the tRF-315 mimic and inhibitor designed by Bioneer is tRF-315 mimic, 5′-GCC CGG CUA GCU CAG UCG GUA GAG CAU GG-3′ and tRF-315 inhibitor, 5′-CCA UGC UCU ACC GAC UGA GCU AGC CGG GC-3′. The control oligonucleotide (siCTR), siRNA against angiogenin (siANG), and siRNA against GADD45A (siGADD45A) were also purchased from Bioneer.

### 2.5. BrdU Incorporation Analysis

The cell proliferation ELISA BrdU kit (Roche) was used according to the manufacturer’s instructions as described previously [20]. Briefly, the cells were seeded in a 96-well plate (5 × 103 cells). The cells were transfected with the tRF-315 mimic for 6 h and then treated with cisplatin for 48 h. After incubation, 10 μM BrdU was added to the cell culture and the cells were incubated for an additional 2 h at 37 °C. After labeling the cells with BrdU, the fixed cells were incubated with anti-BrdU-peroxidase (POD) working solution for 90 min. The anti-BrdU-POD binds to BrdU incorporated in newly synthesized cellular DNA, and the resulting immune complexes were detected by their reaction to the 3,3′,5,5′-Tetramethylbenzidine (TMB) substrate. The absorbance values of the reaction products were quantified by measuring the absorbance at 370 nm and 492 nm using an ELISA reader. The data represent three independent experiments.

### 2.6. Annexin V and Propidium Iodide Staining

To analyze apoptosis, Annexin V apoptosis detection kit I (BD Biosciences, Franklin Lakes, NJ, USA) was used according to the manufacturer’s instructions, as previously described [20]. Briefly, cells (4 × 10^5^ cells) were seeded onto 6-well plates. The cells were then transfected with a tRF-315 mimic or inhibitor for 6 h and then treated with cisplatin for 48 h. Supernatants were removed from culture dishes and adherent cells were detached using trypsin-EDTA. The cells were collected via centrifugation, washed with PBS, and resuspended in 1× binding buffer. Then, 100 μL of the cell suspension was transferred to a 5 mL culture tube and incubated with 5 μL FITC Annexin V and 5 μL of PI for 15 min at room temperature in the dark. Then, 400 μL of 1× binding buffer was added to the 5 mL culture tube. Fluorescence intensity was analyzed using a flow cytometer (BD Biosciences). The graph was quantified as the ratio of the upper right and lower left of the quadrant. The data represent three independent experiments.

### 2.7. Quantitative RT-PCR

Target gene expression was determined as described previously [21]. Complementary DNA was synthesized using total RNA (1 µg) and AccuPower^®^ RT PreMix (Bioneer, Daejeon, Korea). Gene expression levels were measured using SYBR^®^ Green (Sigma-Aldrich) and StepOnePlus™ Real-Time PCR System (Applied Biosystems, Foster City, CA, USA). We determined the expression levels of target genes using standard curves and CT values. The PCR conditions were 95 °C for 3 min, followed by 40 cycles at 95 °C for 30 s, 60 °C for 30 s, and 72 °C for 30 s. The PCR conditions were maintained using a melting curve program (increasing the temperature from 55 °C to 95 °C at a rate of 0.5 °C per 10 s) with continuous fluorescence measurement. ROX dye (Invitrogen) was used as a negative control for the fluorescence measurements. Sequence-specific products were identified by generating a melting curve in which the CT value represented the cycle number at which a fluorescent signal was statistically greater than the background, and relative gene expression was quantified. Expression of *GAPDH*, stable under most experimental conditions, was used to normalize the expression of target genes. The sequence of the primer used for PCR is as follows: *ANG*, 5′-CAG CAC TAT GAT GCC AAA CC-3′ (sense), and 5′-GAT GTC TTT GCA GGG TGA GG-3′ (antisense); *TP53*, 5′-GTC TTT GAA CCC TTG CTT GC-3′ (sense), and 5′-CCA CAA CAA AAC ACC AGT GC-3′ (antisense); *BAX*, 5′-TTT GCT TCA GGG TTT CAT CC-3′ (sense), and 5′-ACA CTC GCT CAG CTT CTT GG-3′ (antisense); *GADD45A*, 5′-GAG AGC AGA AGA CCG AAA GC (sense), and 5′-GGA TGT TGA TGT CGT TCT CG-3′ (antisense).

### 2.8. Western Blotting

Western blotting was performed as previously described to determine protein expression [20]. Cells were transfected with the tRF-315 inhibitor for 6 h and protein concentrations in whole-cell extracts were determined using the Bradford protein assay (Bio-Rad Hercules, CA, USA) with bovine serum albumin (BSA) as the standard. Proteins were denatured, separated using sodium dodecyl sulphate–polyacrylamide gel electrophoresis (SDS-PAGE), and transferred to nitrocellulose membranes. Blots were developed using enhanced chemiluminescence detection (SuperSignal West Pico, Pierce, Rockford, IL, USA) and were quantified by measuring the intensity of light emitted from correctly sized bands under ultraviolet light using a ChemiDoc EQ system and Quantity One software (Bio-Rad, Hercules, CA, USA). Immunoreactive proteins were detected using goat anti-rabbit polyclonal antibodies against phospho-proteins and total-proteins at 1:1000 dilution and 10% SDS-PAGE gel. Total proteins were used as loading controls to normalize the results of western blotting. The details of all antibodies used are listed in Table 1. The data represent three independent experiments.

### 2.9. Analysis of Mitochondrial Membrane Potential

A mitochondrial staining kit (Sigma-Aldrich) was used to measure MMP in prostate cancer cells [21]. Briefly, cells (5 × 10^5^ cells) were seeded into 6-well plates. The cells were transfected with the tRF-315 mimic or inhibitor for 6 h and then treated with cisplatin for 48 h. Supernatants were removed from culture dishes and adherent cells were detached using trypsin-EDTA. The cells were collected via centrifugation, resuspended in staining solution containing 200× JC-1 in 1× staining buffer, and incubated at 37 °C in a CO_2_ incubator for 20 min. The stained cells were collected via centrifugation and washed once with 1× JC-1 staining buffer. After washing, the cell suspension was centrifuged once more and the cells were resuspended in 1 mL staining buffer. Fluorescence intensity was analyzed using a FACSCalibur instrument. The graph was quantified as the lower right/upper right ratio. The data represent three independent experiments.

### 2.10. Cell Cycle Analysis

Cell cycle was analyzed by flow cytometry after RNase A (Sigma-Aldrich) treatment and propidium iodide (PI) staining. Cells (5 × 10^5^ cells) were seeded in 6-well plates and incubated to 70% confluency. Cells were transfected with the tRF-315 mimic or inhibitor for 6 h and then treated with cisplatin for 48 h. Cells were divided into the subG1, G0/G1, S, and G2/M phases. The data represent three independent experiments.

### 2.11. Statistical Analysis

The statistical significance of all results was verified via analysis of variance using the SAS program. A probability value of *p* < 0.05 was considered statistically significant. Data are expressed as the mean ± standard deviation (SD) for three independent experiments.

## 3. Results

### 3.1. tRF-315 Is More Abundant in Prostate Cancer Cells than in Normal Prostate Cells

**Figure 1:**
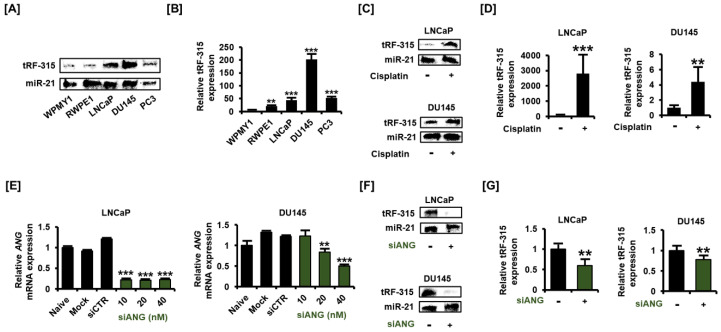
tRF-315 is abundantly expressed in prostate cancer cell lines. (**A**) The expression of tRF-315 in WPMY1, RWPE1, LNCaP, DU145, and PC3 cells was detected by blotting using DIG-labeled probe, splinted ligation, and EDC cross-linking. (**B**) Quantitative PCR was performed to quantify the expression of tRF-315 in WPMY1, RWPE1, LNCaP, DU145, and PC3 cells. (**C**) The expression of tRF-315 in response to cisplatin (20 μM) treatment for 24 h in LNCaP and DU145 cells was detected by blotting. (**D**) Quantitative PCR was performed to quantify the expression of tRF-315 in response to cisplatin (20 μM) treatment for 24 h in LNCaP and DU145 cells. (**E**) The mRNA expression of *ANG* in LNCaP and DU145 cells in response to siANG (10, 20, and 40 nM) for 6 h was measured via quantitative PCR. (**F**) The expression of tRF-315 in response to transfection of siANG (20 nM) for 6 h in LNCaP and DU145 cells was detected by blotting. (**G**) Quantitative PCR was performed to quantify the expression of tRF-315 in response to the transfection of siANG (20 nM) for 6 h in LNCaP and DU145 cells. The expression of tRF-315 was quantified using the 2^−ΔΔCT^ method based on standard curves and cycle threshold (C_T_) values. The data represent three independent experiments. The asterisks indicate significant differences between treated cells and control cells (*** *p* < 0.001 and ** *p* < 0.01).

We first compared the expression of tRF-315 in normal prostate cell lines (WPMY1 and RWPE1) with that in prostate cancer cell lines (LNCaP, DU145, and PC3). The previously described method for the detection of small RNAs, involving the use of the non-radioactive DIG-labeled probe, was used in this study with modifications [19]. MiR-21 was detected together to correct the difference in the amount of RNA when loaded onto urea-polyacrylamide gel. The results of blotting revealed that tRF-315 levels were higher in prostate cancer cells than in normal prostate cells (Figure 1A). To compensate for the inaccuracy of blotting and to accurately quantify the expression of tRF-315 in prostate cell lines, quantitative PCR was performed using the cDNA synthesized after performing a polyadenylation reaction. The expression of tRF-315 was found to be the highest in DU145 cells (202.1 ± 21.3-fold increase compared to WPMY1 cells) among all prostate cell lines (Figure 1B). In LNCaP and PC3 cells, the expression of tRF-315 was found to be higher than that in normal prostate cells. We used LNCaP and DU145 cells to measure changes in the expression of tRF-315 upon cisplatin treatment. Interestingly, tRF-315 expression was found to be elevated following cisplatin treatment in LNCaP cells (Figure 1C). Moreover, cisplatin also induced an increase in tRF-315 expression in DU145 cells, although the increase was not as much as that in LNCaP cells. An increase in tRF-315 expression upon cisplatin treatment in prostate cancer cells was also confirmed by quantitative PCR (Figure 1D). In LNCaP cells, cisplatin increased tRF-315 expression by 2816.7 ± 1215.2-fold (*p* < 0.001) compared to that in control. In DU145 cells, the expression of tRF-315 was increased by about 4.4 ± 1.9-fold (*p* < 0.001) in response to cisplatin. We confirmed the efficacy by measuring the expression of *ANG* after dose-dependent transfection of siANG into LNCaP and DU145 cells. In both cells, siANG concentrations greater than 20 nM significantly inhibited *ANG* expression (Figure 1E). Next, we transfected siANG (20 nM) into LNCaP and DU145 cells to determine whether angiogenin affects the production of tRF-315. The results of blotting showed that the expression of tRF-315 was reduced in LNCaP and DU145 cells following transfection of the cells with siANG, while the expression of miR-21 remained unaffected (Figure 1F). According to the results of quantitative PCR, siANG transfection significantly decreased the expression of tRF-315 in LNCaP and DU145 cells (Figure 1G). The expression of tRF-315 was reduced by 41.0 ± 13.2% (*p* < 0.01) and 22.0 ± 6.8% (*p* < 0.01) in siANG-transfected LNCaP and DU145 cells, respectively. These results suggest that tRF-315 is relatively abundantly expressed in prostate cancer cells than in normal prostate cells and that the expression of tRF-315 may be increased by the cellular stress caused by cisplatin.

### 3.2. Transfection with tRF-315 Mimics Does Not Affect Prostate Cancer Cell Proliferation but Leads to the Induction of Prostate Cancer Cell Death

**Figure 2:**
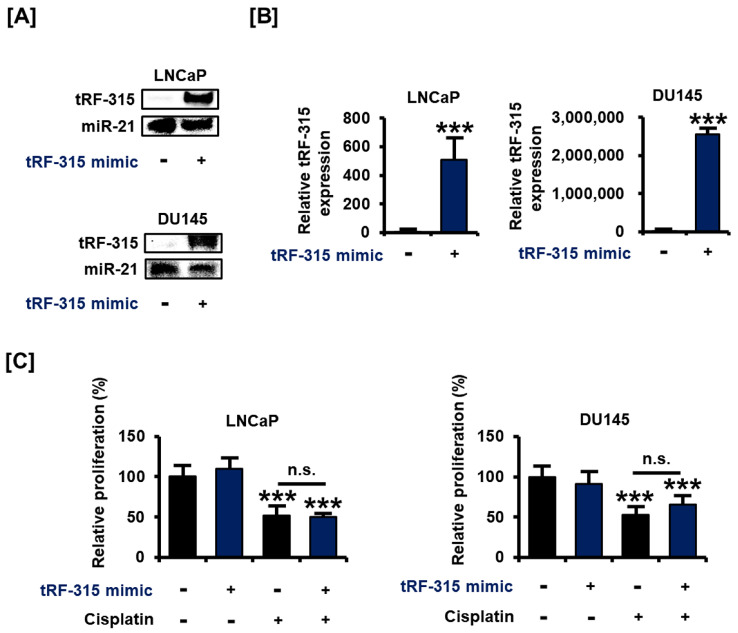
tRF-315 mimic does not affect the proliferation of prostate cancer cells. (**A**) The expression of tRF-315 in response to the transfection of the tRF-315 mimic (20 nM) for 6 h in LNCaP and DU145 cells was detected by blotting. (**B**) Quantitative PCR was performed to quantify the expression of tRF-315 in response to transfection of tRF-315 mimic (20 nM) for 6 h in LNCaP and DU145 cells. The expression of tRF-315 was quantified using the 2^−ΔΔCT^ method based on standard curves and cycle threshold (C_T_) values. (**C**) The change in the proliferation of LNCaP and DU145 cells following transfection with the tRF-315 mimic (20 nM) for 6 h and cisplatin (20 μM) treatment for 24 h was confirmed by BrdU analysis. The value of the control group was expressed as 100%. The data represent three independent experiments. The asterisks indicate significant differences between the treated cells and control cells (*** *p* < 0.001). The symbol ‘n.s.’ indicates that there is no significance between the two groups.

Next, we designed and transfected a tRF-315 mimic to verify the change in the characteristics of the prostate cancer cells under the control of tRF-315. The results of blotting revealed that the tRF-315 mimic increased the intensity of DIG signal for the tRF-315 probe in LNCaP and DU145 cells (Figure 2A). These results were comparable with the results of quantitative PCR (509.1 ± 139.0-fold increase in LNCaP cells and 2,551,495.3 ± 132,817.6-fold increase in DU145 cells) (Figure 2B). We first tested whether the transfection with the tRF-315 mimic affects the proliferative capacity of prostate cancer cells, which was reduced by cisplatin. However, tRF-315 mimics did not affect the proliferation of LNCaP and DU145 cells (Figure 2C).

**Figure 3:**
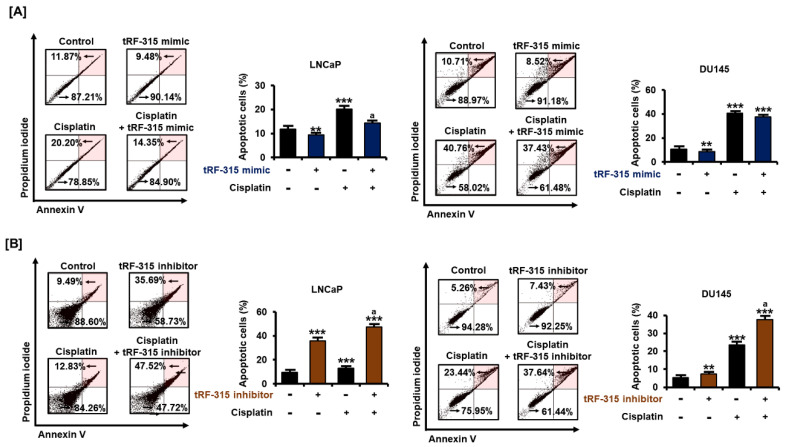
In prostate cancer cells, tRF-315 regulates apoptosis induced by cisplatin. (**A**) Annexin V and propidium iodide-stained cells indicate apoptotic cell death. The percentage of the upper right quadrant was determined to quantify the induction of apoptosis following transfection of cells with tRF-315 mimic (20 nM) for 6 h and cisplatin treatment for 48 h. (**B**) The ratio of the upper right to the lower left quadrants was determined to quantify the induction of apoptosis following the transfection of cells with the tRF-315 inhibitor (20 nM) for 6 h and cisplatin (20 μM) treatment for 48 h. The value of the control group was expressed as 100%. The data represent three independent experiments. The asterisks indicate significant differences between the treated cells and control cells (*** *p* < 0.001 and ** *p* < 0.01). The symbol ‘a’ indicates significant difference compared with cisplatin alone treatment group.

Next, we examined the proportion of apoptotic cells by transfecting the tRF-315 mimic into cells treated with cisplatin to determine whether tRF-315 could affect prostate cancer cell death using annexin V and PI staining. The results revealed that the tRF-315 mimic alone inhibited the apoptosis of LNCaP (9.5 ± 0.3%, *p* < 0.01) and DU145 (8.5 ± 1.1%, *p* < 0.01) cells (Figure 3A). Moreover, the cisplatin-induced increase in apoptosis was alleviated upon transfection of the tRF-315 mimic into the cells. In LNCaP cells, tRF-315 mimic transfection reduced the number of apoptotic cells, compared to the case for the treatment with cisplatin alone. In DU145 cells, the tRF-315 mimic decreased the cisplatin-induced apoptosis. In addition, treatment with the tRF-315 inhibitor alone induced the apoptosis of LNCaP and DU145 cells, which was increased further upon treatment with cisplatin (47.5 ± 1.3% in LNCaP cells and 37.6 ± 1.2% in DU145 cells) (Figure 3B). These results suggest that tRF-315 may be involved in prevention of apoptosis induced by cisplatin, although independent of the pathways that regulate proliferation in prostate cancer cells.

### 3.3. Expression of tRF-315 Is Involved in the Sensitivity to Cisplatin-Induced Apoptotic Pathway in Prostate Cancer Cells

**Figure 4:**
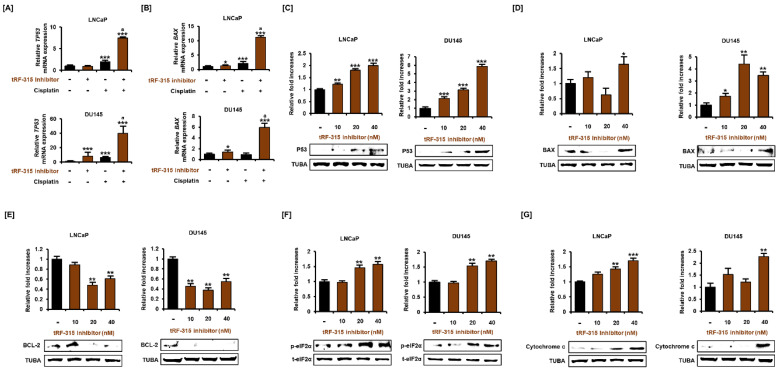
tRF-315 inhibitor induces activation of the apoptotic pathway in prostate cancer cells. The mRNA expression of *TP53* (**A**) and *BAX* (**B**) in response to the tRF-315 inhibitor (20 nM) for 6 h and cisplatin (20 μM) for 24 h was measured via quantitative PCR. The protein expression of P53 (**C**), BAX (**D**), BCL-2 (**E**), phosphorylated eIF2α (**F**), and cytochrome c (**G**) in response to tRF-315 inhibitor (10, 20, and 40 nM) for 24 h was measured by western blotting. The data represent three independent experiments. The asterisks indicate significant differences between the treated cells and control cells (*** *p* < 0.001, ** *p* < 0.01, and * *p* < 0.01). The symbol ‘a’ indicates a significant difference compared with cisplatin alone treatment group.

Next, we investigated whether inhibition of tRF-315 affects the expression of proteins belonging to the apoptotic pathway because tRF-315 inhibitor alone could induce apoptosis in prostate cancer cells. P53 is a central protein responsible for the apoptotic pathway in cancer cells and has been reported to be induced by cisplatin in prostate cancer cells [3]. It has been reported that LNCaP cells express wild-type P53, while DU145 cells express a mutated form of P53 [22]. In LNCaP and DU145 cells, the tRF-315 inhibitor further enhanced the mRNA levels of P53 induced by cisplatin (7.5 ± 0.2-fold increase in LNCaP cells and 40.1 ± 7.7-fold increase in DU145 cells, compared to the control) (Figure 4A). In addition, the mRNA level of *BAX*, a downstream protein of P53 and a pro-apoptotic protein, was also increased by the tRF-315 inhibitor in the presence of cisplatin in LNCaP and DU145 cells (11.2 ± 0.3-fold increase in LNCaP cells and 5.9 ± 0.7-fold increase in DU145 cells, compared to the control) (Figure 4B). Western blotting analysis revealed that tRF-315 inhibitors dose-dependently increased the expression of P53 in DU145 (5.9 ± 0.2-fold at 40 nM, *p* < 0.001) and LNCaP (2.0 ± 0.1-fold at 40 nM, *p* < 0.001) cells (Figure 4C). The expression of BAX protein was increased (1.6 ± 0.2-fold increase in LNCaP cells and 3.5 ± 0.3-fold increase in DU145 cells at 40 nM), while the expression of the anti-apoptotic protein BCL-2 was decreased by the tRF-315 inhibitor (38.7 ± 3.6% reduction in LNCaP cells and 45.2 ± 4.8% reduction in DU145 cells at 40 nM) (Figure 4D,E). It has been reported that phosphorylation of eIF2α protects against stress-induced cell death via the inhibition of P53 and BAX stabilization and their translocation to the mitochondria [23,24]. Conversely, excessive activation of eIF2α in cancer cells also symbolizes endoplasmic reticulum (ER) stress-mediated apoptosis [25]. In LNCaP and DU145 cells, the tRF-315 inhibitor induced phosphorylation of eIF2α (1.6 ± 0.1-fold increase in LNCaP cells and 1.7 ± 0.1-fold increase in DU145 cells at 40 nM), suggesting that inhibition of tRF-315 in itself can lead to cellular stress, which leads to activation of the apoptotic pathway in prostate cancer cells (Figure 4F). Finally, we identified the expression of cytochrome c, which is involved in mitochondrial-dependent apoptosis. We found that the tRF-315 inhibitor significantly increased the expression of cytochrome c in LNCaP (1.7 ± 0.1-fold at 40 nM, *p* < 0.001) and DU145 (2.3 ± 0.1-fold at 40 nM, *p* < 0.01) cells (Figure 4G). These results suggest that the inhibition of tRF-315 can lead to P53- and mitochondria-dependent apoptosis in prostate cancer cells.

### 3.4. tRF-315 Alleviates Mitochondrial Dysfunction Induced by Cisplatin in Prostate Cancer Cells

**Figure 5:**
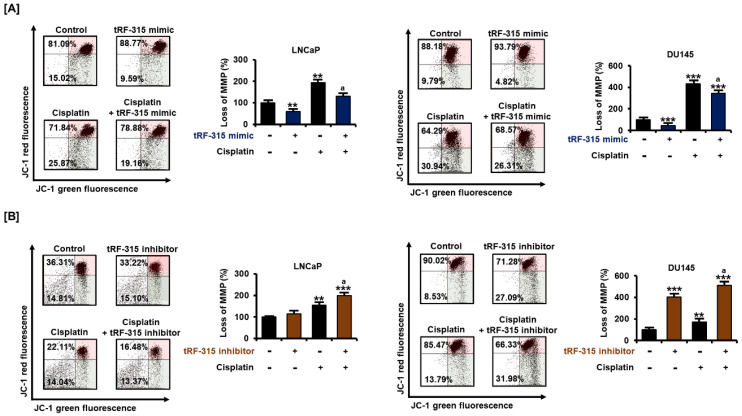
Effects of tRF-315 on mitochondrial membrane potential elevated upon cisplatin treatment in LNCaP and DU145 cells. (**A**) Change in mitochondrial membrane potential in response to transfection with tRF-315 mimic (20 nM) for 6 h and cisplatin (20 μM) treatment for 48 h in LNCaP and DU145 cells was analyzed by JC-1 staining. (**B**) Change in mitochondrial membrane potential in response to transfection with the tRF-315 inhibitor (20 nM) for 6 h and cisplatin (20 μM) treatment for 48 h in LNCaP and DU145 cells was analyzed by JC-1 staining. The lower right/upper right values were calculated for quantification. The value of the control group was expressed as 100%. The data represent three independent experiments. The asterisks indicate significant differences between the treated cells and control cells (*** *p* < 0.001 and ** *p* < 0.01). The symbol ‘a’ indicates a significant difference compared with cisplatin alone treatment group.

Next, we confirmed whether tRF-315 was involved in mitochondrial dysfunction during the apoptotic process in prostate cancer cells. Cisplatin treatment significantly reduced the mitochondrial membrane potential (MMP) in LNCaP and DU145 cells (Figure 5). However, upon transfection of tRF-315 in both cells, the cisplatin-induced mitochondrial depolarization was mitigated. In LNCaP cells, tRF-315 mimic reduced the MMP loss induced by cisplatin by about 32.5 ± 4.2% (*p* < 0.05). In DU145 cells, tRF-315 mimic reduced the cisplatin-induced MMP loss by 20.3 ± 4.6% (*p* < 0.05). In contrast, tRF-315 inhibitor further enhanced MMP loss elevated by cisplatin treatment in LNCaP (2.0 ± 0.1-fold compared to control, *p* < 0.001) and DU145 (5.1 ± 0.2-fold compared to control, *p* < 0.001) cells. These results suggest that tRF-315 may have a protective effect against cisplatin-induced mitochondrion-dependent apoptosis.

### 3.5. As a Target of tRF-315, GADD45A Is Involved in Cell Cycle Regulation by Cisplatin in Prostate Cancer Cells

**Figure 6:**
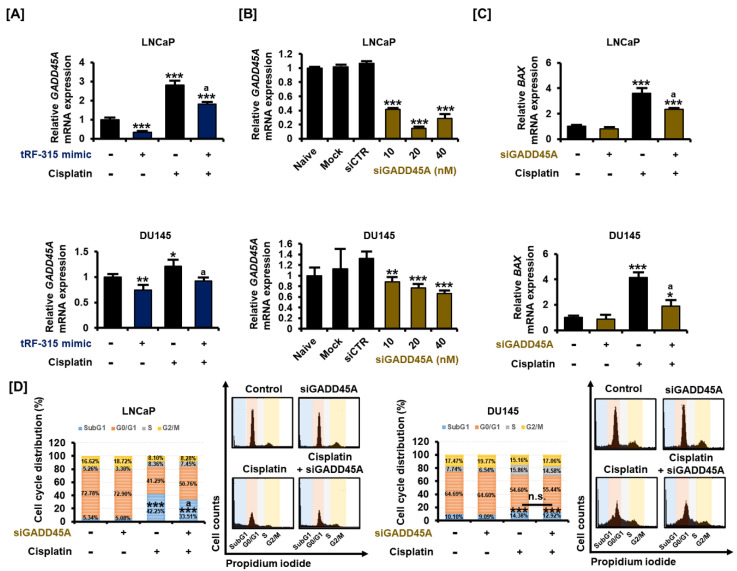
tRF-315 targets *GADD45A*, which regulates the cell cycle in prostate cancer cells. (**A**) The mRNA expression of *GADD45A* in LNCaP and DU145 cells in response to tRF-315 mimic (20 nM) for 6 h and cisplatin (20 μM) for 24 h was measured via quantitative PCR. (**B**) The mRNA expression of *GADD45A* in LNCaP and DU145 cells in response to siGADD45A (10, 20, and 40 nM) for 6 h was measured via quantitative PCR. (**C**) The mRNA expression of *BAX* in in LNCaP and DU145 cells response to siGADD45A (20 nM) for 6 h and cisplatin (20 μM) for 24 h was measured via quantitative PCR. (**D**) Cell cycle distribution was confirmed through propidium iodide staining. The effect of siGADD45A (20 nM) and cisplatin (20 μM) on the cell cycle was measured based on the number of cells in the sub G1, G1, S, and G2/M phases. The data represent three independent experiments. The asterisks indicate significant differences between the treated cells and control cells (siCTR) (*** *p* < 0.001, ** *p* < 0.01, and * *p* < 0.01). The symbol ‘a’ indicates a significant difference compared with cisplatin alone treatment group. The symbol ‘n.s.’ indicates that there is no significant difference between the two groups.

Similar to miRNAs, tRFs are also involved in the transcriptional regulation of target genes. We selected genes that tRF-315 can target based on a miRNA database. Among them, we confirmed the tRF-315-induced change in the expression of the growth arrest and DNA damage 45 alpha (*GADD45A*) gene, which is stimulated by P53 and well known as the tumor suppressor gene. Cisplatin increased the expression of *GADD45A* in LNCaP (2.8 ± 0.2-fold, *p* < 0.001) and DU145 (1.2 ± 0.1-fold, *p* < 0.05) cells, and this effect was significantly alleviated by the tRF-315 mimic, as verified by quantitative PCR (Figure 6A). We confirmed that the expression of *GADD45A* decreased after the dose-dependent transfection of siGADD45A into LNCaP and DU145 cells (Figure 6B). GADD45A mainly maintains genome stability by regulating the cell cycle and induces BAX expression during apoptosis, which results in mitochondrial dysfunction. We found that the cisplatin-mediated increase in the expression of *BAX* was alleviated by siGADD45A in LNCaP (35.1 ± 0.1%, *p* < 0.05) and DU145 (54.3 ± 2.8%, *p* < 0.05) cells (Figure 6C). Moreover, cell cycle analysis revealed that the proportion of cells in the subG1 phase observed following cisplatin treatment was decreased upon transfection of siGADD45A in LNCaP, but not in DU145 cells (Figure 6D). In LNCaP cells, the proportion of cells in the subG1 phase increased to 42.25 ± 2.4% upon treatment with cisplatin and decreased to 33.51 ± 1.8% upon transfection of the cells with siGADD45A (*p* < 0.05). In DU145 cells treated with cisplatin, siGADD45A transfection led to a decrease in the proportion of cells in the subG1 phase from 14.38 ± 0.7% to 12.92 ± 0.5%, but this decrease was not statistically significant. These results suggest that tRF-315 could protect prostate cancer cells by inhibiting the expression of GADD45A during cisplatin-induced mitochondrion-dependent apoptosis.

## 4. Discussion

Androgen deprivation therapy is an important method for the treatment of prostate cancer; however, it can lead to the progression of castration-resistant prostate cancer. To date, no effective treatment has been developed for patients who are resistant to androgen-deprivation therapy. To develop an effective prostate cancer treatment strategy, we need to study the molecular mechanisms underlying the resistance to chemotherapy. Cisplatin is a standard chemotherapy agent widely used for various types of cancer, including ovarian and cervical cancer, as well as prostate cancer [26]. Cisplatin damages DNA in cancer cells, disrupting normal transcription and cellular functions. High concentrations of cisplatin, however, may affect the normal organs in the body. This limitation forces clinicians to administer an appropriate concentration of cisplatin, which may lead to the development of resistance in patients with advanced cancer [27,28]. In this study, we aimed to find novel molecular targets that can regulate the cytotoxicity of cisplatin in prostate cancer cells. Recently, to find new therapeutic molecular targets for drugs, genetic profiling has been performed to investigate drug resistance in prostate cancer [29,30,31]. P53 is a tumor suppressor responsible for regulating cell death, cell cycle, and DNA repair. In many malignant tumors, P53 is deleted or mutated. Moreover, cisplatin increases P53 expression in prostate cancer cells and inhibits the expression of androgen receptor and prostate-specific antigen (PSA), along with the suppression of cell proliferation [3]. Expression of *BAX* in prostate cancer cells also tends to be similar to that of P53 in response to cisplatin treatment [4]. We confirmed that the expression of P53 and BAX increased in response to cisplatin at both the mRNA and protein levels in prostate cancer cells, as reported previously [3,32].

sncRNAs are also involved in the development of platinum-based drug resistance in prostate cancer [33]. In prostate cancer cells, miR-205 enhances cisplatin toxicity through the induction of cleaved caspase-9 and cytochrome c [9]. In addition, a miR-425-5p mimic suppresses the expression of cyclin D1 and targets GSK3β, to increase the sensitivity of prostate cancer cells to cisplatin [11]. Conversely, the miR-17-92 cluster promotes the growth of prostate cancer cells and causes cisplatin resistance [10]. In prostate cancer, miR-32-5p regulates the expression of BCL-2-interacting killer (BIK), a proapoptotic protein belonging to the BCL-2 family, and induces resistance to cisplatin-induced apoptosis [33].

However, little is known about whether other types of sncRNAs, besides miRNAs, influence the effects of cisplatin in cancer cells. Studies in the past decade have raised the possibility that tRFs could be considered as potential indicators for cancer diagnosis [34]. However, little is known about whether tRFs have physiological functions associated with cancer progression. Fortunately, it has been proven that in prostate cancer, several tRFs can regulate the proliferation of prostate cancer cells. Moreover, among the different types of cancer, prostate cancer is relatively well known for changes in the profile of tRFs compared to the normal cells. tRF-1001, derived from a tRNA^Ser^ precursor, is associated with the proliferation of prostate cancer cells [12]. Honda and his colleagues have shown that the knockdown of tRF^Lys^ inhibits the proliferation of LNCaP cells [18]. In their study, they suggested that angiogenin, whose transcription is promoted by the androgen receptor, leads to the production of tRFs. We confirmed that tRF-315 derived from tRNA^Lys^ does not affect the proliferation of prostate cancer cells, including LNCaP cells, but it was indirectly confirmed that tRF-315 could be generated by the activity of angiogenin. The production of tRFs involves the activity of various enzymes, such as angiogenin, dicer, and RNase Z, depending on the target site being cleaved [35,36]. We found that angiogenin knockdown in prostate cancer cells reduced the expression of tRF-315. However, elucidation of the exact site of the action of angiogenin on tRNA^Lys^ requires further studies. In addition, there are doubts as to why tRF-315 does not affect the proliferation of prostate cancer cells. Although several literatures suggest that only one characteristic of proliferation and apoptosis can be regulated in cells, the mechanism of the specific effect of tRF-315 on apoptosis needs to be confirmed [37].

Magee et al. found via clinical statistical analysis that short 5′-tRFs (18–20 nt) are frequently expressed in the normal prostate samples, whereas 5′-tRFs of 29 and 30 nt are highly expressed in prostate cancer samples [17]. The results of this study, with 29 nucleotide-long tRF-315 showing relatively high expression in prostate cancer cell lines, complement the results of this previous report. Moreover, it has been reported that the ratio of tRFs derived from tRNA^Lys^ (tRF-315) and tRNA^Phe^ (tRF-544) can be a good indicator of the progression-free survival of patients with prostate cancer [16]. It has been reported that tRNA cleavage increases under stress conditions in cells [37,38]. In this study, cisplatin significantly increased the expression of tRF-315 in prostate cancer cells. This suggests that the production of tRF-315 in response to cisplatin increased and may have had a protective effect. We suggest that tRF-315 not only has a protective effect against cisplatin in prostate cancer cells, but that the inhibition of tRF-315 alone can lead to apoptosis and mitochondrial dysfunction. This suggests that tRF-315 can serve as a therapeutic target, as well as a predictive indicator, for prostate cancer. Recent evidence has shown that tRFs can regulate cell growth in a P53-dependent manner [39,40]. Detailed mechanisms underlying these phenomena require further studies, but our study has revealed that tRF-315 can also regulate the expression of P53 and apoptotic pathway-related proteins in prostate cancer cells. Moreover, the induction of cytochrome c by tRF-315 suggests that mitochondria may be targeted for intracellular regulation by tRFs. LNCaP and DU145 cells used in this study express wild-type and mutant forms of P53, respectively. However, the sensitivity to cisplatin does not differ significantly depending on the status of P53 [3]. Rather, another evidence revealed that the growth-inhibiting effect of cisplatin was more sensitive in DU145 cells than in LNCaP cells, unlike other anticancer drugs [22]. Both LNCaP and DU145 cells respond to tRF-315 inhibitors, resulting in the increased expression of P53, and consequently, apoptosis, suggesting that like cisplatin, tRF-315 can also function in a P53-dependent and P53-independent manner [3].

tRFs are also involved in the regulation of gene transcription. For instance, tRF^Glu^ targets Kruppel-like factor (KLF) to inhibit mRNA expression and regulate adipogenesis [41]. In breast cancer, tRFs compete with the RNA-binding site of the YBX1 protein to inhibit cell growth [42]. Target prediction analysis based on the database suggested that tRF-315 can target multiple genes. Among them, GADD45A is well known for inhibiting cell growth in various types of cancers, including prostate cancer [43,44,45]. *GADD45A* is a downstream target gene that is transcriptionally regulated by P53 [46]. However, the tumor-suppressing effects of GADD45A are observed in cell lines with both the wild-type P53 and P53-negative status [47]. Previous evidence that the upregulation of *GADD45A* in DU145 cells enhances sensitivity to docetaxel suggests that the anticancer agents may also function via GADD45A, independent of functional P53 [43]. The primary function of GADD45A in cancer cells is to regulate the cell cycle and inhibit cell growth. In squamous cell carcinoma cells, knockdown of *GADD45A* inhibits apoptosis, with the reduction of *BAX* gene expression and induction of *BCL-2* gene expression [48]. In addition, *GADD45A* methylation in DNA in serum has been suggested as a biomarker for prostate cancer in combination with PSA, since it can clinically distinguish prostate cancer [49]. Several therapeutic aids that can be used to treat prostate cancer target GADD45A [50]. Fucoxanthin, a member of the carotenoid family extracted from brown algae, causes an increase in the expression of *GADD45A* in LNCaP cells and leads to arrest of the cells in the G1 phase [51]. Isoliquiritigenin, a chalcone found in licorice, induces G2/M phase arrest and apoptosis in prostate cancer cells, with an increase in GADD45A expression [52]. When the expression of *GADD45A*, which was targeted by tRF-315, was inhibited, the cisplatin-induced increase in the proportion of cells in the subG1 phase in prostate cancer was reduced. This suggests that the inhibition of *GADD45A* may prevent apoptosis in prostate cancer cells, implying the cell growth regulatory function of GADD45A. Moreover, the suppressive effect of siGADD45A on the cisplatin-induced increase in the proportion of only LNCaP cells in the subG1 phase suggests that the inhibitory effect of cisplatin on cell growth may be dependent on the P53–GADD45A axis. In addition, the increase in *GADD45A* expression by cisplatin is more sensitive in LNCaP cells than in DU145 cells. Considering that LNCaP cells have a wild-type P53, the mechanism of GADD45A induction by cisplatin is assumed to be P53-dependent.

## 5. Conclusions

**Figure 7:**
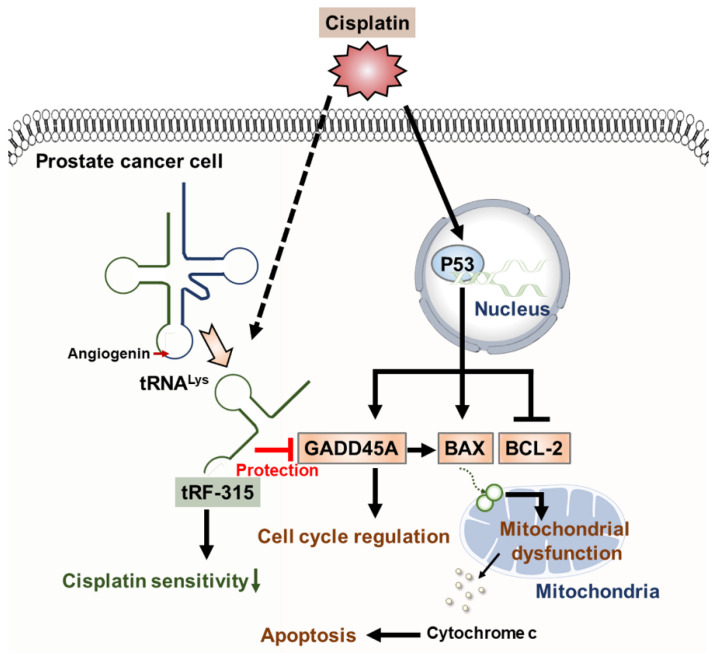
Schematic diagram describing the effects of tRF-315 on prostate cancer cells treated with cisplatin. Cisplatin induces the expression of GADD45A and BAX and inhibits the expression of BCL-2 mediated by P53 in prostate cancer cells. BAX leads to mitochondrial-dependent apoptosis. Meanwhile, in response to cisplatin, endogenous tRNA^Lys^ are cleaved to tRF-315 due to the activity of angiogenin. *GADD45A* is the target gene for tRF-315. tRF-315 alleviates mitochondrial dysfunction and prevents apoptosis caused by cisplatin in prostate cancer cells. As a result, tRF-315 reduces the sensitivity of prostate cancer cells to cisplatin. Therefore, it is estimated that inhibition of tRF-315 can increase the sensitivity of cisplatin by regulating the expression of apoptosis-related proteins and inducing mitochondrial mediated apoptosis in prostate cancer cells.

Taken together, this study suggests that tRF-315, derived from tRNA^Lys^, may contribute to the reduced sensitivity to cisplatin by targeting genes including *GADD45A*, as illustrated in Figure 7. To our knowledge, this study is the first to identify the mechanism by which tRFs induce apoptosis in prostate cancer cells. In addition, we confirmed that tRFs can interfere with mitochondrial morphology. Since the study of the role of tRFs in cancer, including prostate cancer, is still in its infancy, it is speculated that the accumulation of these findings could reveal the potential use of tRFs in the diagnosis and treatment of various cancers in the future.

## Figures and Tables

**Table 1:** List of primary antibodies used in Western blot analysis.

Primary Antibodies	Dilution	Supplier	Catalog Number
P53	1:1000	Cell Signaling Technology	2527
BAX	1:1000	Cell Signaling Technology	2772
BCL-2	1:1000	Cell Signaling Technology	2876
Phosphor-eIF2α	1:1000	Cell Signaling Technology	3398
eIF2α	1:1000	Cell Signaling Technology	5324
Cytochrome c	1:1000	Cell Signaling Technology	11940
TUBA	1:1000	Santa Cruz	sc-5286

## Data Availability

Data available on request due to restrictions eg privacy or ethical.
